# Dual-low spectral CT pulmonary angiography: a comparative study of image quality, radiation dose, and iodine intake with evaluation of pulmonary embolism detection

**DOI:** 10.3389/fmed.2026.1797846

**Published:** 2026-06-15

**Authors:** Hui Li, Li Zhou, Yipu Mao, Qiyao Zou, Meihai Xu, Jianhui Ou, Changjian Lao, Xinning Gong

**Affiliations:** 1 Department of Radiology, The First People’s Hospital of Nanning, Guangxi, China; 2 Department of Radiology, The Second People’s Hospital of Nanning, Guangxi, China

**Keywords:** CT pulmonary angiography, CT radiation dose, dual-flow injection, iodine contrast media, spectral CT

## Abstract

**Background:**

CT pulmonary angiography (CTPA) is widely recognized as the first-line imaging modality for pulmonary embolism (PE) because of its rapid and accurate characteristics. However, the radiation exposure and risk of contrast-induced acute kidney injury associated with conventional CTPA have motivated continuous efforts to develop “dual-low” protocols that achieve both low radiation dose and low iodine contrast dose.

**Purpose:**

To evaluate the feasibility of a dual-low CT pulmonary angiography (CTPA) protocol integrating spectral CT, complete dual-flow injection, and low iodine dose (10 mL), and to verify three primary endpoints: (1) reduction of effective radiation dose (ED); (2) reduction of iodine intake; (3) superior subjective image quality (3-point scale) under low-dose conditions. Secondary endpoint: added value of iodine density maps for visualizing perfusion defects.

**Methods:**

Prospective randomized controlled trial. 160 suspected pulmonary embolism (PE) patients randomized to experimental group (*n* = 80, 10 mL iodine + spectral CT) or control group (*n* = 80, 30 mL iodine + conventional CT). Two blinded radiologists evaluated subjective image quality (3-point scale), measured CT attenuation, signal-to-noise ratio (SNR), contrast-to-noise ratio (CNR), effective radiation dose (ED), iodine intake, and analyzed iodine maps.

**Results:**

Subjective score higher in experimental group (2.69 ± 0.44 vs. 2.33 ± 0.44, *p* < 0.001; Kappa = 0.767). Objective parameters lower in experimental group (all *p* < 0.001) but CNR (24.08 ± 7.15) > diagnostic threshold (CNR > 10). ED reduced by 41.2% (2.51 ± 0.59 mSv vs. 4.27 ± 0.94 mSv, *p* < 0.001); iodine intake reduced by 66.7% (3.5 vs. 10.5 g). PE detection rate: 53.7% (43/80) vs. 38.7% (31/80), *p* = 0.081. Iodine maps clearly showed perfusion defects.

**Conclusion:**

The dual-low spectral CTPA protocol achieved 41.2% radiation reduction, 66.7% iodine reduction, and superior subjective image quality. Iodine density maps provide complementary functional information. This protocol is a safe and effective alternative for CTPA.

## 1 Introduction

Pulmonary embolism (PE) is a potentially fatal cardiovascular emergency, and early accurate diagnosis is essential to improve patient outcomes (1). CTPA has become the first-line imaging method for PE because of its high sensitivity (83%) and specificity (78–100%) (2). However, the radiation exposure associated with conventional CTPA (effective dose ED typically 3–5 mSv) and the risk of contrast-induced acute kidney injury remain concerns (3). To address these issues, traditional “dual-low” protocols mainly reduce tube voltage (e.g., to 100 kVp) to lower radiation dose. This strategy, however, increases image noise due to decreased X-ray penetration. To maintain intraluminal iodine concentration, higher iodine doses or more concentrated contrast media are often required, creating a dilemma in which reducing radiation makes it difficult to reduce iodine load. A systematic review and meta-analysis published in 2025 (including 35 studies) evaluated low tube voltage combined with reduced contrast volume in CTPA and showed that this strategy can lower effective radiation dose by 50–80% while preserving diagnostic image quality (4). Spectral CT offers a new technical solution to this contradiction. By acquiring dual-energy data with rapid kV switching, it generates low-keV virtual monoenergetic images that exploit the photoelectric effect near the iodine K-edge (33.2 keV), thereby markedly increasing iodine contrast and providing a theoretical basis for obtaining diagnostic image quality under extremely low iodine load (5). Recently, Zhang et al. (6) demonstrated that deep learning reconstruction can improve image quality of 40-keV monoenergetic images in low-dose dual-energy CTPA. Shen et al. (7) reported that deep learning reconstruction combined with a dual-low dose CTPA protocol reduces radiation and contrast dose while improving image quality. The complete dual-flow injection technique, which mixes iodine contrast with saline in real time and at a fixed ratio, optimizes contrast rheology in central vessels, reduces beam-hardening artifacts in the superior vena cava and right heart, and improves diagnostic comfort (8).

The multi-mode post-processing functions of spectral CT allow combined morphological and functional evaluation of PE. Virtual non-contrast (VNC) images can replace true non-contrast scans, directly reducing radiation dose (9); iodine density maps can visually depict pulmonary parenchymal perfusion, enabling a “morphology-function” one-stop assessment of PE (9).

Therefore, we conducted a prospective randomized controlled trial to evaluate the overall feasibility of a novel CTPA protocol integrating spectral CT, complete dual-flow injection, and a low iodine dose (10 mL). The three primary endpoints were: (1) reduction of effective radiation dose; (2) reduction of iodine intake; and (3) superior subjective image quality under dual-low conditions (superiority design). The secondary endpoint was to evaluate the added value of iodine density maps for depicting perfusion defects associated with PE.

## 2 Materials and methods

### 2.1 Study design and patient population

This single-center prospective randomized controlled trial was funded by a self-raised research grant from the Guangxi Zhuang Autonomous Region Health Commission (contract No. Z-A20240996). The study protocol was approved by the Medical Ethics Committee of Nanning First People’s Hospital (approval No. LW2026-DECISION-002), and all enrolled patients gave written informed consent. We included adult patients (≥18 years) with clinically suspected acute PE (presenting with dyspnea, chest pain, hemoptysis, or syncope) who were scheduled for CTPA between October 2023 and December 2025. Exclusion criteria were severe renal insufficiency (eGFR < 30 mL/min/1.73 m^2^), known allergy to iodinated contrast media, pregnancy, or lactation.

#### 2.1.1 Sample size estimation

The primary endpoint for sample size calculation was the subjective image quality score (3-point Likert scale), using a superiority design. The estimation parameters were derived from a published SCI article (10) that used a conventional CTPA protocol similar to our control group (high-pitch scanning, 50 mL iodine contrast) and reported that >90% of images were rated as good to excellent on a 3-point Likert scale. Based on those data, we estimated a mean score of approximately 2.30 ± 0.45 for the conventional protocol. We anticipated that the dual-low spectral CTPA protocol would increase the score by 0.40 points (to 2.70), a superiority margin based on the image improvement observed in a pilot study (*n* = 20) using 50-keV monoenergetic spectral CT images. With a two-sided *α* of 0.05 and a power (1-*β*) of 90%, the sample size formula for two independent means yielded 66 patients per group. Accounting for approximately 15% attrition, we finally set 80 patients per group, for a total of 160 patients.

#### 2.1.2 Randomization

Simple randomization was used. A researcher not involved in patient recruitment, scanning, or image evaluation generated a computer-randomized number table to assign eligible patients in a 1:1 ratio to the experimental group (dual-low spectral CTPA) or the control group (conventional dose CTPA). Allocation was concealed in sequentially numbered, sealed, opaque envelopes. After informed consent was signed, a CT technologist opened the envelope in order and performed the assigned scanning and injection protocol. The groups were balanced with 80 patients each. No statistically significant differences were observed between groups in age, sex, or body mass index (all *p* > 0.05), indicating successful randomization.

### 2.2 Study endpoints

#### 2.2.1 Primary endpoints

To verify the synergistic advantages of the experimental protocol in three dimensions:

- Radiation dose reduction: effective radiation dose lower than that in the control group (preset reduction ≥30%).
- Iodine intake reduction: total iodine intake lower than that in the control group (preset reduction ≥50%).
- Subjective image quality superiority: under dual-low conditions, the subjective image quality score (3-point scale) superior to that of the control group (superiority design, preset superiority margin ≥0.30 points).

#### 2.2.2 Secondary endpoint

To evaluate the added value of spectral CT iodine density maps in the diagnosis of PE, including analysis of the correspondence between perfusion defects shown on iodine maps and embolus morphology (complete vs. incomplete) and its clinical significance for improving diagnostic confidence and providing functional information.

### 2.3 CT scanning and iodine contrast injection protocols

All examinations were performed on a GE CT scanner (256-detector, 16-cm wide-body, Revolution).

#### 2.3.1 Experimental group (dual-low spectral CTPA)

- Scan mode: Spectral imaging (GSI) with instantaneous switching between 80 kVp and 140 kVp, and automatic tube current modulation (noise index NI = 9.0 HU). Bolus tracking was used (trigger threshold 100 HU, delay 3 s). After scanning, VNC images were used instead of true non-contrast scans.
- Iodine contrast protocol: Complete dual-flow injection. Non-ionic iodinated contrast (iohexol, 350 mgI/mL) 10 mL and saline 20 mL were mixed in real time at a 1:2 ratio using a dual-syringe power injector (CTpanion D, Hengrui Medical) and injected at 4.5 mL/s into an antecubital vein, followed by a 20 mL saline flush at the same rate.

#### 2.3.2 Control group (conventional dose CTPA)

- Scan mode: A true non-contrast scan was performed first (100 kVp, automatic tube current modulation, NI = 9.0 HU), followed by contrast-enhanced scanning using a conventional spiral mode with the same parameters and bolus tracking (trigger 100 HU, delay 3 s).
- Iodine contrast protocol: Conventional single-flow injection. Iohexol (350 mgI/mL) 30 mL was injected at 4.5 mL/s, followed by a 20 mL saline flush.

**Figure 1:**
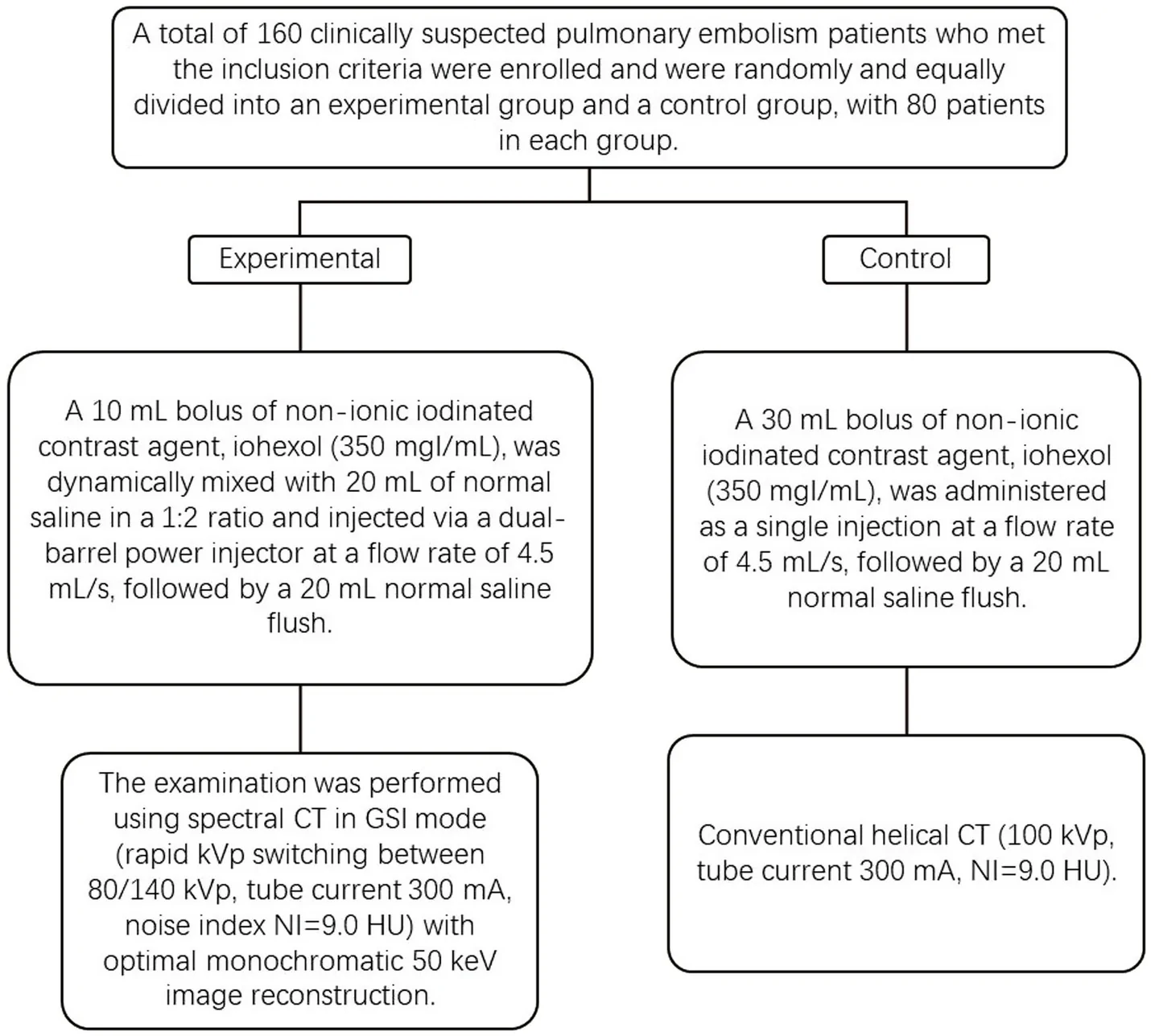
Comparison of dual-flow and conventional injection protocols. The iodine contrast volume in the experimental group (10 mL) is lower than that in the control group (30 mL).

**Figure 2:**
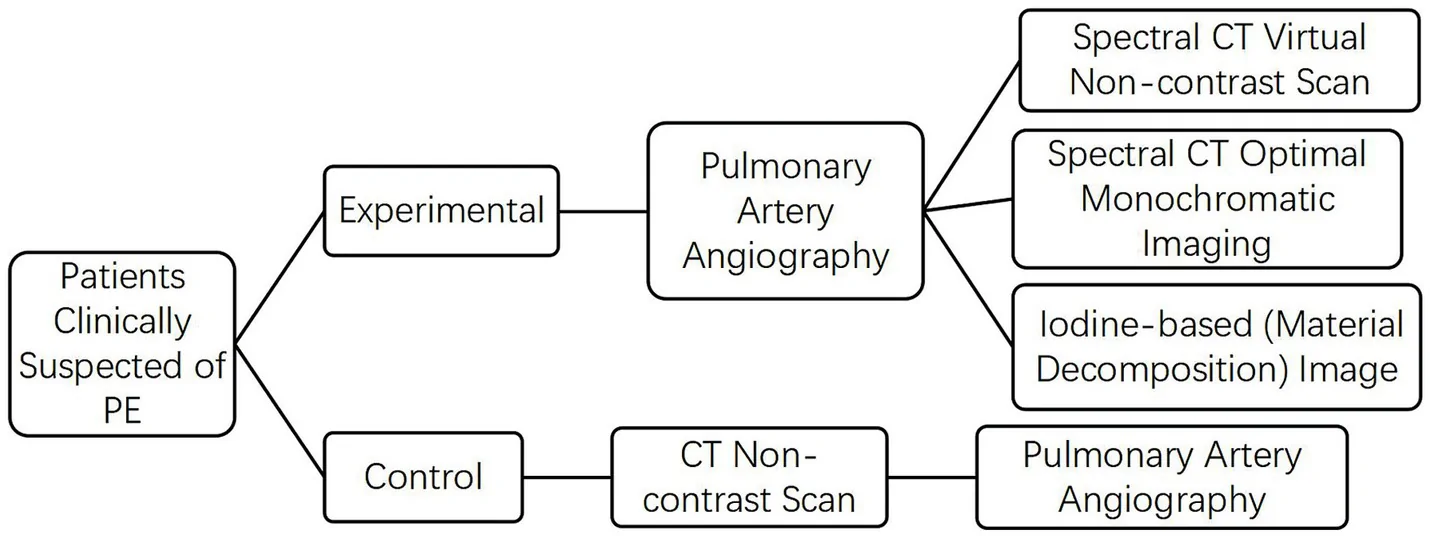
Comparison of the different scanning protocols. By using spectral CT virtual non-contrast imaging, the experimental group avoided an additional true non-contrast scan, reducing radiation dose and simplifying the workflow.

**Figure 3:**
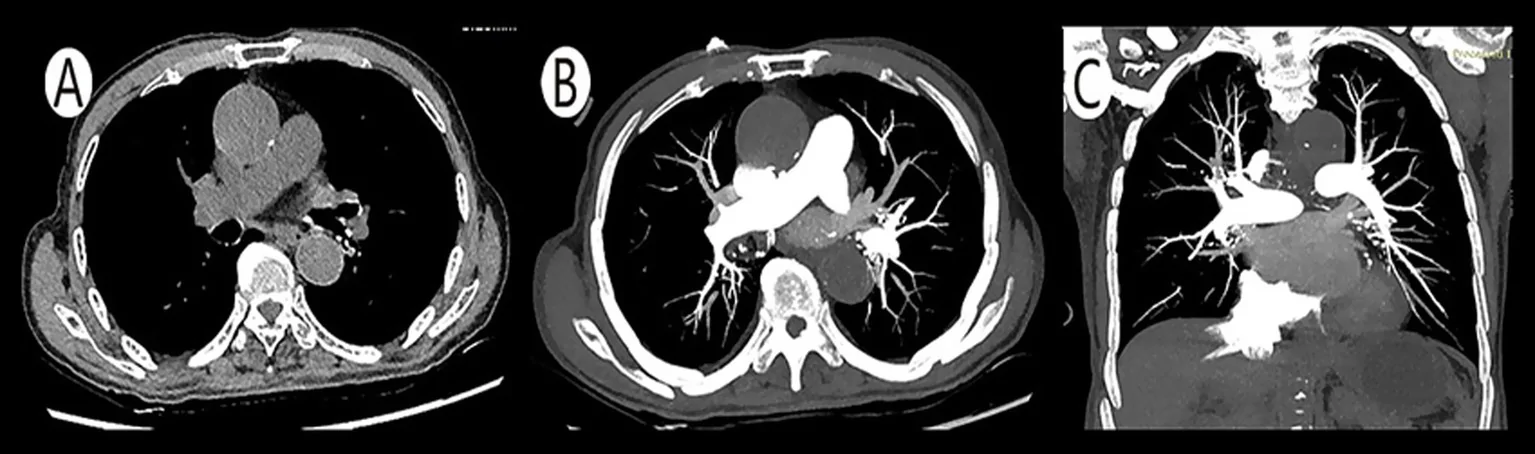
Conventional dose CTPA (control group). Control group used “non-contrast + contrast-enhanced” mode (**A**: non-contrast). Embolism detection relied mainly on contrast-enhanced MIP images **(B,C)** for morphological evaluation, limiting detection of small distal pulmonary arteries (below 7th–9th order).

**Figure 4:**
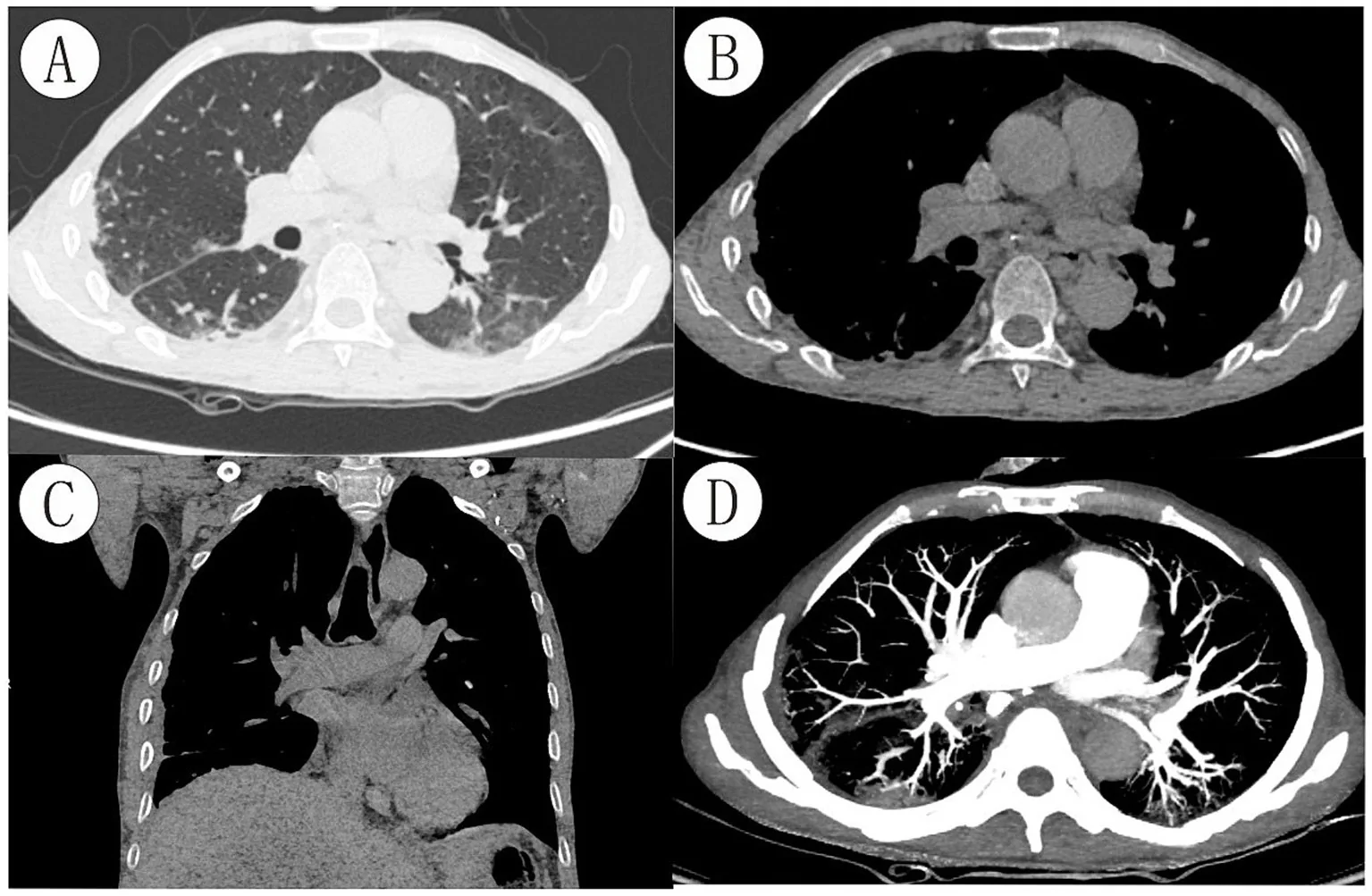
Dual-low spectral CTPA images (experimental group). Experimental group used spectral CT and dual-flow injection (10 mL iodine). **(A–C)** virtual non-contrast (VNC) replacing true non-contrast; **(D)** 50-keV optimal monoenergetic MIP image clearly showing peripheral pulmonary arteries.

**Figure 5:**
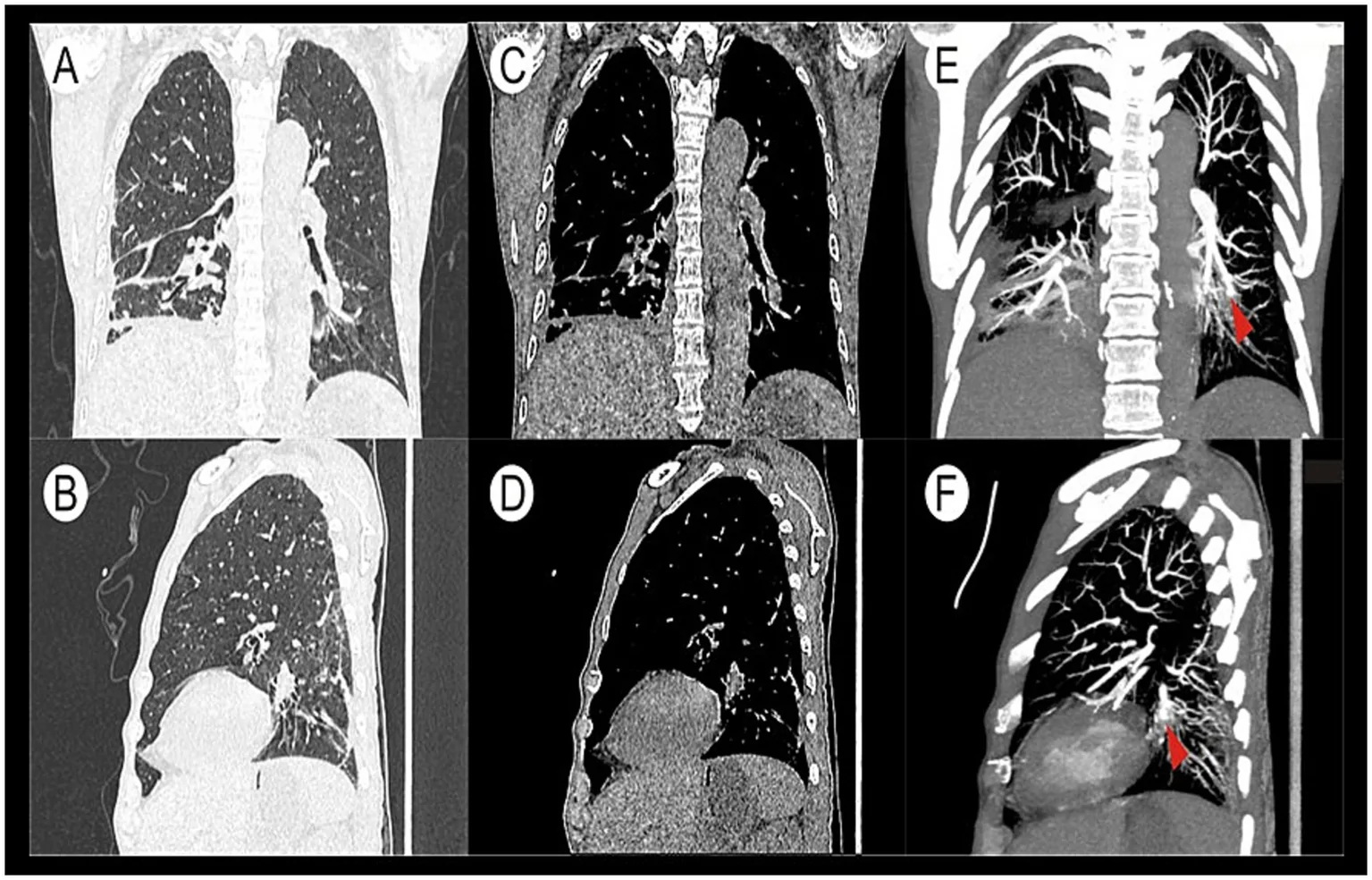
Example case from the experimental group. A 62-year-old male with suspected PE. Spectral CTPA with 10 mL iodine, VNC used instead of true non-contrast **(A–D)**, lung parenchyma well visualized. 50-keV grayscale MIP image **(E,F)** showing suspected emboli in the left lower lobe anterior and posterior basal segment arteries (red arrows, 5th order vessels).

Both groups covered the area from the lung apex to the diaphragm (see Figures 1–5).

### 2.4 Image analysis and quality assessment

Raw data were transferred to a GE AW 4.7 workstation for unified analysis.

#### 2.4.1 Selection of the optimal monoenergetic keV level

To determine the optimal monoenergetic level for image quality evaluation, we used the following method: based on data from 20 patients (pilot group) who underwent spectral CTPA for suspected PE but were not formally enrolled in the main study, we reconstructed 40, 50, 60, and 70 keV monoenergetic images using GSI Viewer software. A radiologist blinded to clinical information measured pulmonary artery CT attenuation, background noise (standard deviation of CT attenuation in the pectoralis major muscle), and calculated CNR at the level of the main pulmonary artery for all 20 patients. The keV level with the highest mean CNR was defined as the optimal monoenergetic level. Analysis showed that 50 keV images had the highest mean CNR. Although 40 keV provided higher iodine signal, its higher image noise led to a lower CNR than at 50 keV. Therefore, considering both CNR and noise, 50 keV was selected as the optimal monoenergetic level. The 20 pilot patients had complete height and weight records, with a BMI range of 19–26 kg/m^2^ (mean 23.4 ± 1.3 kg/m^2^), consistent with the formal study population. We prespecified that 50-keV monoenergetic images would be used as the standard for all subsequent image quality assessments in the experimental group (see Table 1).

**Table 1:** Comparison of CNR at different monoenergetic levels (40–70 keV) in the main pulmonary artery in the pilot group.

Monoenergetic level (keV)	Number of patients (*N*)	Mean CNR (mean ± SD)	*p* value (vs. 50 keV)
40 keV	20	22.99 ± 5.12	< 0.001
50 keV	20	28.18 ± 5.74	
60 keV	20	20.95 ± 4.15	< 0.001
70 keV	20	20.29 ± 4.24	< 0.001

Data in this table are from the pilot study used to determine scanning parameters, not from the 80 patients in the formal study. Compared with 50 keV: 40 keV t = −4.23, *p* < 0.001; 60 keV t = 5.67, *p* < 0.001; 70 keV t = 6.12, *p* < 0.001.

#### 2.4.2 Objective image quality analysis

##### 2.4.2.1 Experimental group

Raw spectral data were reconstructed with a standard kernel into 1.0-mm slice thickness and interval. Based on the optimal monoenergetic level (50 keV) determined in section 1.4.1, corresponding monoenergetic images were reconstructed using GSI Viewer software for subsequent evaluation.

##### 2.4.2.2 Control group

Raw conventional spiral data were reconstructed with the same standard kernel into 1.0-mm slices.

##### 2.4.2.3 Virtual non-contrast (VNC) image generation

VNC images were generated from the arterial-phase spectral data of the experimental group using a material-decomposition VNC algorithm (iterative reconstruction level ASiR-V 40%) in the GSI Volume Viewer software on the GE AW 4.7 workstation, with reconstruction parameters identical to those for the experimental group.

##### 2.4.2.4 Objective image quality measurements

For the experimental group, CT attenuation (HU) was measured in the main pulmonary artery, left and right pulmonary arteries, interlobar arteries, and segmental arteries. ROIs were circular: vascular ROIs were 20–30 mm^2^ placed centrally in the lumen, avoiding vessel walls and calcifications; background noise ROI was placed in the pectoralis major muscle at the same level (area ~50 mm^2^), avoiding fat. The mean CT value and its standard deviation (SD, representing background noise) of the pectoralis major muscle were also measured. SNR and CNR were calculated using the individual background noise SD (standard deviation of CT attenuation within the same ROI placed in the pectoralis major muscle) as the denominator: SNR = (arterial CT value)/(noise SD) and CNR = (arterial CT value−muscle CT value)/(noise SD).

##### 2.4.2.5 Subjective image quality assessment

Two radiologists with 10 and 8 years of chest imaging experience, respectively, independently and blindly evaluated all images. The evaluation procedure was as follows:

- Reading conditions: Images were viewed on dedicated medical diagnostic workstations (professional medical displays, routinely calibrated) in a standard reading room with constant soft ambient light.
- Blinding: All scanning parameters, patient information, and group allocation were hidden; images were presented only with random numbers.
- Reading order: Images from both groups were completely randomized and presented to the readers, who were unaware of batch or group assignment.
- Scoring criteria: 3-point Likert scale: 3 (excellent) – low image noise, vessels clearly and sharply depicted, high diagnostic confidence; 2 (acceptable) – moderate noise, vessels identifiable, meeting basic diagnostic requirements; 1 (poor) – severe noise, vessel boundaries blurred, low diagnostic confidence.
- Disagreement resolution: Disagreements were resolved by joint reading and discussion to reach a consensus, which was used for final analysis. Inter-observer agreement was assessed using Cohen’s Kappa coefficient.

### 2.5 Diagnostic criteria for pulmonary embolism on CTPA

All CTPA images were independently reviewed by two senior chest radiologists (with 15 and 12 years of experience, respectively) who were blinded to group allocation. A positive diagnosis of PE was defined as the presence of a definite intraluminal filling defect on transverse images and multiplanar reformations (MPR). In case of disagreement, a third more senior radiologist arbitrated to reach a consensus.

It should be noted that we used CTPA imaging consensus as the reference standard for filling defects; this standard itself is the test being evaluated, not an independent external gold standard (e.g., clinical follow-up or digital subtraction angiography). Therefore, this study can only compare the positive detection rates between the two protocols and cannot evaluate true diagnostic sensitivity, specificity, or accuracy, and this design has incorporation bias.

### 2.6 Radiation dose and iodine intake calculation

Dose-length product (DLP) for each scan was obtained from the dose report. Effective dose (ED) was calculated according to the European guidelines for quality criteria in CT: ED = DLP × k (chest conversion factor k = 0.014 mSv·mGy^−1^·cm^−1^). Total iodine intake (g) = iodine concentration (350 mgI/mL) × injected volume (mL) / 1,000.

### 2.7 Statistical analysis

SPSS Statistics 25.0 was used. Continuous variables were tested for normality (Shapiro–Wilk). Normally distributed variables are expressed as mean ± SD and compared using independent samples t-test; non-normally distributed variables are expressed as median (interquartile range) [M(IQR)] and compared using Mann–Whitney U test. Categorical variables are expressed as frequencies (percentages) [*n*(%)] and compared using χ^2^ test. For the primary endpoints, subjective image quality scores and ED were compared using independent t-test; total iodine intake, being fixed by protocol with no within-group variation, was described only. For secondary endpoints, objective image quality parameters were compared using independent t-test, and PE detection rates were compared using χ^2^ test. Inter-observer agreement for subjective scores was assessed using Cohen’s Kappa (Kappa > 0.75 considered good agreement). All tests were two-sided, and *p* < 0.05 was considered statistically significant.

#### 2.7.1 Missing data handling

A total of 34 patients (10 in the experimental group, 24 in the control group) did not have height or weight recorded because they were brought by stretcher or admitted through the emergency department. For BMI comparisons, we used complete case analysis, i.e., only patients with complete height and weight data (70 in the experimental group, 56 in the control group). To verify robustness, a sensitivity analysis was performed using multiple imputation (MICE, 5 imputations). After imputation, the difference in BMI remained non-significant (*p* = 0.161), and the conclusions for the primary outcome measures (subjective image quality, ED, etc.) were unchanged, indicating that missing data did not materially affect the study conclusions.

## 3 Results

### 3.1 Baseline patient characteristics

Experimental group (*n* = 80, with complete height-weight data in 70): Age range 40–65 years, mean 52.94 ± 8.21 years; 45 males, 35 females; BMI range 19–26 kg/m^2^, mean 23.11 ± 1.50 kg/m^2^.

Control group (*n* = 80, with complete height-weight data in 56): Age range 40–65 years, mean 53.62 ± 8.75 years; 42 males, 38 females; BMI range 19–26 kg/m^2^, mean 23.42 ± 1.21 kg/m^2^.

There were no statistically significant differences in age, sex, or BMI between the two groups (all *p* > 0.05). The difference in BMI was also not significant (*p* = 0.157) (see Table 2).

**Table 2:** Baseline patient characteristics.

Characteristic	Experimental group (*n* = 80)	Control group (*n* = 80)	Statistic	*p* value
Age (years)	52.94 ± 8.21	53.61 ± 8.75	t = −0.497	0.620
Sex, *n* (%)			χ^2^ = 0.225	0.635
Male	45 (56.2)	42 (52.5)		
Female	35 (43.8)	38 (47.5)		
BMI (kg/m^2^)	23.11 ± 1.50 (*n* = 70)	23.42 ± 1.21 (*n* = 56)	t = −1.423	0.157

Ten patients in the experimental group and 24 in the control group did not have height/weight measured because they were on stretchers or emergency admissions; BMI comparisons are based on complete case analysis. Sensitivity analysis with multiple imputation confirmed that missing data did not affect conclusions.

### 3.2 Image quality assessment

#### 3.2.1 Subjective image quality

The final subjective image quality score was higher in the experimental group than in the control group (2.69 ± 0.44 vs. 2.33 ± 0.44, t = −4.836, *p* < 0.001). Inter-observer agreement was good (Kappa = 0.767, *p* < 0.001) (see Table 3).

**Table 3:** Subjective image quality scores.

Group	Reader A	Reader B	Final score
Experimental	2.68 ± 0.47	2.68 ± 0.47	2.69 ± 0.44
Control	2.34 ± 0.48	2.33 ± 0.47	2.33 ± 0.44
t value	4.527	4.710	5.120
*p* value	< 0.001	< 0.001	< 0.001

#### 3.2.2 Objective image quality

All objective parameters (CT attenuation, SNR, CNR) were lower in the experimental group than in the control group (all *p* < 0.001). However, the mean CNR in the experimental group (24.08 ± 7.15) far exceeded the internationally accepted diagnostic threshold (CNR > 10), indicating that 50-keV monoenergetic images can meet clinical diagnostic requirements even with an extremely low iodine load (see Table 4).

**Table 4:** Objective image quality parameters.

Group	Main pulmonary artery (MPA)	Left pulmonary artery (LPA)	Right pulmonary artery (RPA)	Lobar/segmental arteries	SNR	CNR
Experimental	364.78 ± 78.79	346.83 ± 75.38	349.14 ± 71.44	332.40 ± 67.16	30.15 ± 6.82	24.08 ± 7.15
Control	496.98 ± 163.43	470.99 ± 138.43	477.84 ± 153.86	437.41 ± 142.77	37.45 ± 12.03	32.41 ± 13.22
t value	−6.52	−7.05	−6.79	−5.95	−4.71	−4.96
*p* value	< 0.001	< 0.001	< 0.001	< 0.001	< 0.001	< 0.001

SNR and CNR were calculated using individual background noise SD (standard deviation of CT attenuation in the pectoralis major muscle) as the denominator. Because of large standard deviations, medians are also provided: experimental group MPA median 362.50 HU (IQR 315.20–412.30); control group 485.60 HU (IQR 398.50–592.30).

### 3.3 Radiation dose and iodine intake

#### 3.3.1 Radiation dose

ED was 41.2% lower in the experimental group (2.51 ± 0.59 mSv vs. 4.27 ± 0.94 mSv, *p* < 0.001).

#### 3.3.2 Iodine intake

Iodine intake was reduced by 66.7% (3.5 g vs. 10.5 g, fixed by injection protocol, no within-group variation) (see Table 5).

**Table 5:** Radiation dose and iodine intake.

Group	Effective radiation dose (ED, mSv)	Iodine intake (g)
Experimental	2.51 ± 0.59	3.5
Control	4.27 ± 0.94	10.5
Reduction	41.2%	66.7%
t value	−15.32	
*p* value	< 0.001	

Reduction = (control mean – experimental mean)/control mean × 100%. Iodine intake was fixed by protocol, so SD, t, and *p* are not applicable. The difference (3.5 g vs. 10.5 g) is due to the protocol design. The experimental group also avoided true non-contrast scanning by using VNC, further reducing radiation dose.

### 3.4 Pulmonary embolism detection

According to the CTPA imaging criteria in section 1.5, the detection of filling defects was as follows:

- Experimental group (*n* = 80): 43 patients positive for filling defects, positive detection rate 53.7%.
- Control group (*n* = 80): 31 patients positive, positive detection rate 38.7%.

The difference in positive detection rates was not statistically significant (χ^2^ = 3.042, *p* = 0.081). This result only reflects the numerical difference in detection of filling defects in this patient population and cannot be interpreted as superiority or inferiority in true diagnostic performance. The numerically higher detection rate in the experimental group warrants further investigation with larger sample sizes.

### 3.5 Multi-mode post-processing applications of spectral CT

#### 3.5.1 Virtual non-contrast and optimal monoenergetic imaging

**Figure 6:**
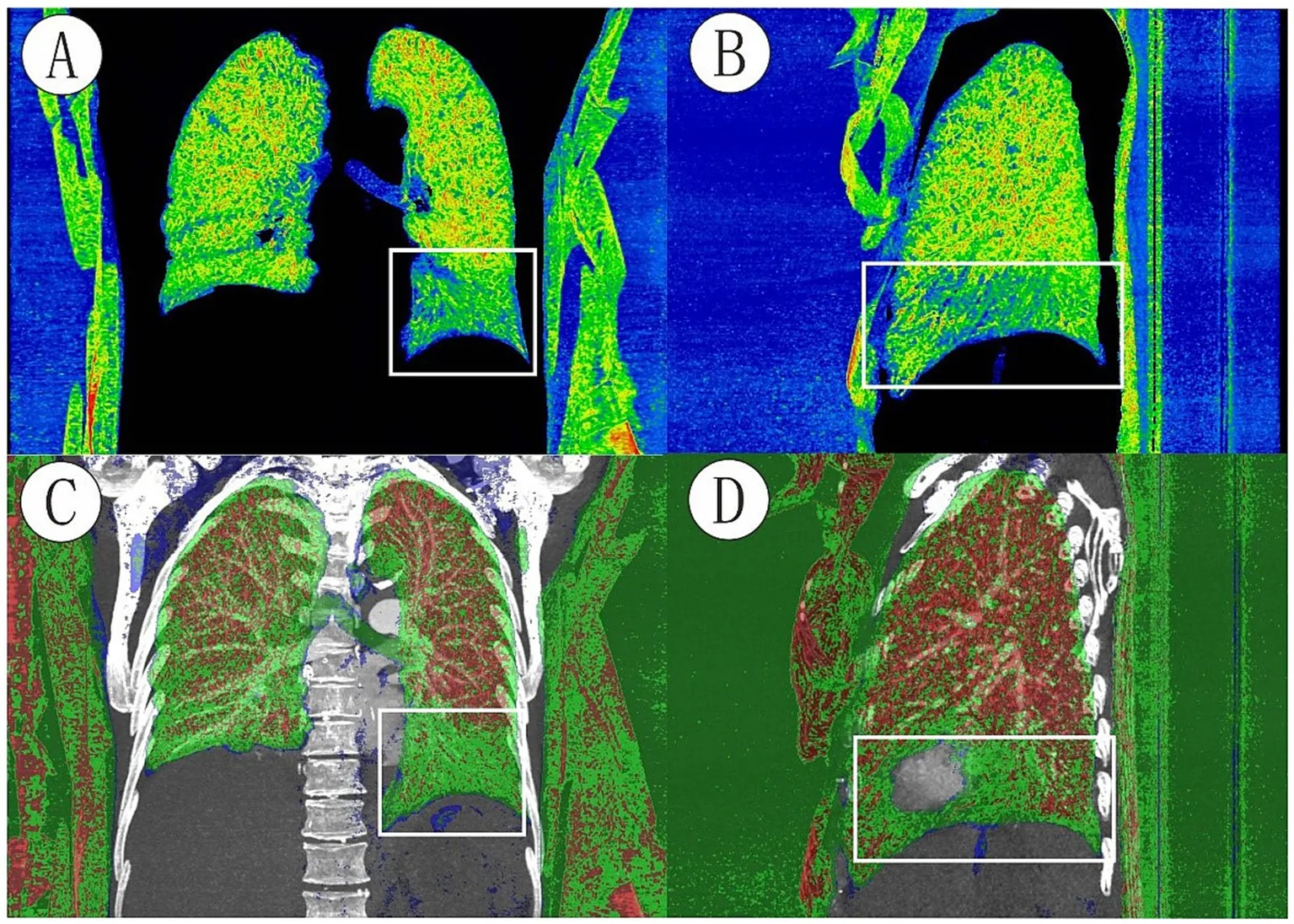
Same case as Figure 5. **(A,B)** Iodine density maps showing wedge-shaped areas of reduced iodine uptake (white boxes); **(C,D)** fused images directly demonstrating morphology-function correspondence.

Maximum intensity projection (MIP) reconstructions were performed for both groups to optimize display of the pulmonary arteries. MIP images clearly showed the complete course, luminal continuity, and morphology, location, and extent of filling defects. To further improve visual recognition, pseudo-color rendering (color map) was applied to the MIP images of the experimental group, enhancing tissue contrast through color gradients and making vessel-background boundaries and subtle density differences easier to identify. Iodine density maps (perfusion images, see Figure 6) visually depicted perfusion defects, providing functional information complementary to morphological assessment.

#### 3.5.2 Anatomical distribution of PE and iodine map analysis in the experimental group

The experimental group used iodine density maps to achieve “morphology-function” integrated evaluation of spectral CT, capturing the complete pathophysiological chain from embolus morphology to perfusion consequences, providing more comprehensive information for identifying various types of PE (including eccentric types) (see Tables 6, 7).

**Table 6:** Anatomical distribution of PE (experimental group *N* = 43, control group *N* = 31).

Anatomical site	Experimental group (*n*)	Experimental group (%)	Control group (*n*)	Control group (%)
Main pulmonary artery	2	4.7	1	3.2
Left/right main pulmonary artery	11	25.6	11	35.5
Interlobar artery	15	34.9	12	38.7
Segmental artery	23	53.5	18	58.1

Percentages are based on total positive cases in each group. A single patient could have emboli at multiple sites, so percentages may sum to >100%.

**Table 7:** Morphology and perfusion defect types on iodine density maps (experimental group, *N* = 43).

Category	Number (*n*)	Percentage (%)
Complete emboli (with wedge-shaped perfusion defect)	5	11.6
Incomplete emboli	38	88.4
Eccentric filling defect (with irregular/mosaic perfusion defect)	10	23.2
Central filling defect	28	65.1*

Of the 28 central filling defects, 23 had a clear corresponding perfusion defect (82.1% of central defects, 53.5% of all positive cases). Percentages are based on 43 positive cases. Iodine density maps are based on spectral CT data. The control group, which underwent conventional CT, could not generate spectral data. The number of complete emboli is small (*n* = 5), so conclusions need validation in larger samples.

## 4 Discussion

In recent years, researchers have continuously attempted to optimize radiation dose and iodine contrast volume while maintaining CTPA image quality. Rajiah et al. (5) and Zhou et al. (9) showed that spectral CT monoenergetic imaging can increase iodine contrast efficiency, providing a theoretical basis for reducing iodine load while maintaining diagnostic image quality. The complete dual-flow injection technique can optimize contrast rheology and reduce beam-hardening artifacts in the superior vena cava and right heart (8). However, these techniques have mostly been studied separately, and their synergistic effects have not been systematically validated. Therefore, we attempted to explore an integrated protocol combining low-iodine-dose spectral scanning with the complete dual-flow injection technique to see whether the synergy could provide a new path for “dual-low” goals.

By systematically comparing CNR across 40–70 keV, we found that 50-keV monoenergetic images provided the highest CNR and were thus selected as the optimal monoenergetic level for this study. The integrated protocol combined spectral CT (50-keV monoenergetic imaging), complete dual-flow injection, and a low iodine dose (10 mL). The results showed that the protocol successfully achieved the three prespecified goals: first, ED was 41.2% lower than in the control group (2.51 ± 0.59 mSv vs. 4.27 ± 0.94 mSv, *p* < 0.001); second, iodine intake was reduced by 66.7% (3.5 g vs. 10.5 g); third, although all objective parameters (CT value, SNR, CNR) were lower in the experimental group, the subjective image quality score was higher (2.69 ± 0.44 vs. 2.33 ± 0.44, *p* < 0.001).

The phenomenon that objective parameters were lower but subjective scores higher has several possible explanations. First, 50-keV monoenergetic images from spectral CT amplify iodine contrast at the physical level. Although the absolute CT attenuation is lower than that on conventional 100-kVp images, the relative difference between iodine and surrounding tissues is more prominent. This high contrast may give radiologists greater confidence when evaluating vessel boundaries and small branches. Second, the dual-flow injection technique, by mixing contrast and saline in real time, theoretically optimizes contrast rheology and may reduce beam-hardening artifacts in the superior vena cava and right atrium (8), thereby improving image clarity and diagnostic comfort. However, we did not perform quantitative artifact analysis (e.g., measuring standard deviation of attenuation in the superior vena cava or attenuation heterogeneity), so these explanations need further validation. In addition, the visual characteristics of 50-keV monoenergetic images and conventional 100-kVp mixed-energy images are inherently different. Although the evaluation was blinded, experienced radiologists might unintentionally recognize the different reconstruction modes, introducing potential observer expectation bias. The separation between subjective and objective quality metrics is likely multifactorial, and the exact mechanisms require future studies using dedicated artifact quantification methods (e.g., measuring the standard deviation of CT attenuation in the right atrium/superior vena cava).

Spectral CT functional imaging has potential in the diagnosis of PE. First, VNC images generated from experimental group spectral data successfully replaced true non-contrast scans, consistent with the report by Zhou et al. (9). This technique directly avoids the additional radiation from a true non-contrast scan (typically 0.6–0.8 mSv), bringing the radiation burden of CTPA closer to a screening level; it also simplifies the workflow, shortens examination time, and improves patient tolerance and cooperation. Tagliati et al. (11) also demonstrated that reducing radiation dose to below 1 mSv can still provide diagnostically acceptable images in ultra-low-dose chest CT, echoing our radiation optimization goal. Moreover, our protocol aligns with the “morphology-function” integrated evaluation concept advocated by Zhang et al. (9). In the experimental group, MIP images served as the primary basis for morphological diagnosis of PE, and iodine density maps served as an adjunct, showing perfusion defects corresponding to emboli in 53.7% of positive cases, providing additional functional information to aid diagnostic confidence. Iodine density maps clearly display the lung parenchymal perfusion defects caused by pulmonary emboli, achieving an integrated assessment of morphological localization and functional perfusion. Lee et al. (12) further showed that dual-energy CT with iodine density maps and VNC can effectively differentiate calcified pulmonary thrombi from simple filling defects, suggesting that iodine map analysis may not only be used to show perfusion defects but also potentially for thrombus composition characterization in the future. The functional information provided can increase diagnostic confidence for PE and enable rapid acquisition of hemodynamic information in emergency settings, offering additional decision-making support beyond morphology.

### 4.1 Impact of patient body habitus on optimal monoenergetic keV selection

The 50-keV optimal level was determined based on the mean CNR in the pilot patients (BMI range 19–26 kg/m^2^, mean ~23.5 kg/m^2^). The optimal monoenergetic keV level may vary with patient body habitus. In larger patients, low-keV (e.g., 40–50 keV) images suffer from increased noise due to decreased X-ray penetration, potentially attenuating the theoretical iodine contrast advantage; in leaner patients, low-keV images better exploit the iodine signal enhancement. Recent studies provide references: Krasniqi et al. (13) found that dual-energy CTPA can safely reduce contrast volume by 25% in normal-weight patients. All patients in this study had a BMI range of 19–26 kg/m^2^ (mean ~23.5 kg/m^2^), which falls into the normal to overweight category, and we did not include obese patients (BMI ≥ 30 kg/m^2^). Therefore, the 50-keV choice is appropriate for this body habitus range. For patients with BMI ≥ 30 kg/m^2^, the efficacy of this protocol needs further validation.

The 41.2% radiation dose reduction achieved by this protocol has cumulative benefits for patients requiring repeated follow-up CTPA; the 66.7% reduction in iodine intake directly lowers the risk of contrast-induced acute kidney injury (3), potentially benefiting more patients with impaired renal function. Yoon et al. (14) reported similar reductions in a dual-low dual-energy liver CT study in high-risk HCC patients (ED reduction ~40%, iodine reduction ~50%), indicating that the dual-low strategy is feasible and generalizable across different anatomical sites and disease settings. The systematic review and meta-analysis by van Stiphout et al. (15) noted that deep learning reconstruction can significantly improve image noise and density measurement accuracy in low-dose CT, providing a theoretical basis for integrating deep learning reconstruction into our protocol to further improve objective image quality.

## 5 Conclusion

The proposed dual-low spectral CTPA protocol successfully achieved the three prespecified primary endpoints: a 41.2% reduction in effective radiation dose, a 66.7% reduction in iodine intake, and superior subjective image quality compared with the conventional protocol. The secondary endpoint confirmed that iodine density maps can provide functional information on perfusion defects corresponding to emboli. This protocol represents a safe and effective alternative for CTPA.

### 5.1 Limitations and future directions

This study has several limitations. First, the experimental protocol was a “package” intervention containing three technical components: spectral imaging, dual-flow injection, and low iodine dose. While this integrated design successfully demonstrated the overall superiority of the new protocol over the conventional one, it cannot attribute the observed positive results strictly to any single component. Future studies using a factorial design, randomizing each component independently, could quantify the individual contributions and interactions.

Second, there is an inherent difference in image reconstruction parameters between groups: the experimental group used 50-keV monoenergetic images, while the control group used conventional 100-kVp mixed-energy images. This is due to the different physical principles of the two scanning modes and is not a design flaw. However, this difference may affect the absolute comparison of objective image quality parameters. The aim of this study was to evaluate the overall clinical value of the integrated protocol (radiation dose, iodine intake, diagnostic performance), not to make a pure technical comparison under identical conditions. Future studies could simulate conventional mixed-energy images on a spectral CT platform to allow more direct comparisons.

Third, the single-center design and moderate sample size may limit generalizability. Larger, multi-center prospective validation is needed to confirm the robustness of our findings across different patient populations and scanner models. Additionally, using artificial intelligence for automated keV selection and individualized iodine injection rate optimization remains a direction for future personalization and refinement of this protocol. Finally, the number of complete emboli in the iodine map analysis was small (*n* = 5), so conclusions regarding complete emboli need validation in larger samples.

### 5.2 Reference standard bias

This study used CTPA imaging consensus as the reference standard for filling defects, which is itself the test being evaluated rather than an independent external gold standard (e.g., clinical follow-up or digital subtraction angiography). This design has incorporation bias. Therefore, we can only compare positive detection rates between the two protocols and cannot evaluate true diagnostic sensitivity, specificity, or accuracy. The clinical significance of the numerically higher detection rate in the experimental group (53.7% vs. 38.7%) remains unclear. Because of the lack of an independent reference standard, this numerical difference cannot be directly interpreted as superiority or inferiority in true diagnostic performance, and future studies using clinical follow-up or other independent reference standards are needed. This study was not designed as a non-inferiority trial, nor did it prespecify a non-inferiority margin; therefore, the non-significant difference in detection rates (*p* = 0.081) cannot be interpreted as evidence of equivalence or non-inferiority in diagnostic performance.

Furthermore, although we speculated in the Discussion that the dual-flow injection technique might improve subjective image quality by reducing beam-hardening artifacts, we did not perform any quantitative or semi-quantitative artifact assessment (e.g., measuring standard deviation of CT attenuation or attenuation heterogeneity in the superior vena cava or right atrium). Therefore, this speculation lacks direct data support. The paradox between subjective and objective quality metrics may also be explained by the high-contrast visual characteristics of spectral images or observer expectation bias. Future studies should incorporate predefined artifact assessment metrics to clarify the underlying mechanisms.

## Data Availability

The original contributions presented in the study are included in the article/supplementary material, further inquiries can be directed to the corresponding author.
