# Correction: Dual-low spectral CT pulmonary angiography: a comparative study of image quality, radiation dose, and iodine intake with evaluation of pulmonary embolism detection

**DOI:** 10.3389/fmed.2026.1972492

**Published:** 2026-08-28

**Authors:** Hui Li, Li Zhou, Yipu Mao, Qiyao Zou, Meihai Xu, Jianhui Ou, Changjian Lao, Xinning Gong

**Affiliations:** 1 Department of Radiology, The First People’s Hospital of Nanning, Guangxi, China; 2 Department of Radiology, The Second People’s Hospital of Nanning, Guangxi, China

**Keywords:** CT pulmonary angiography, CT radiation dose, dual-flow injection, iodine contrast media, spectral CT

Authors Hui Li and Li Zhou were erroneously omitted as equal contributing authors.

The original version of this article has been updated.

